# Editorial: Understanding the complexity of child sexual abuse in child and adolescent psychiatry: Progress, gaps and challenges

**DOI:** 10.3389/fpsyt.2023.1147922

**Published:** 2023-02-06

**Authors:** Jordan Sibeoni, Miriam Rassenhofer, Anne Révah-Levy

**Affiliations:** ^1^ECSTRRA Team, UMR-1153, Inserm, Université Paris Cité, Paris, France; ^2^Service Universitaire de Psychiatrie de l'Adolescent, Argenteuil Hospital Centre, Argenteuil, France; ^3^Department of Child and Adolescent Psychiatry/Psychotherapy, University of Ulm, Ulm, Germany; ^4^Department of Child and Adolescent Psychiatry/Psychotherapy, Competence Center Child Abuse and Neglect, Kompetenzzentrum Kinderschutz in der Medizin Baden-Württemberg, University of Ulm, Ulm, Germany

**Keywords:** CSA, child sexual abuse (CSA) disclosure, qualitative study, clinical vignette, forensic psychiatry

Child sexual abuse (CSA) is a major public health issue worldwide that requires innovative research to cover the many aspects to address for reaching a better understanding of this complex issue at different levels—individual, relational, familial, socio-cultural, and forensic. This Research Topic aimed to show the need for a plurality of research approaches and designs. It gathered seven articles that illustrate the diversity and heterogeneity addressing CSA both clinically and theoretically. Diversity and heterogeneity are also reflected in the multitude of methods—clinical vignette, qualitative synthesis, epidemiological approach, or clinical trials—as well as the focuses and themes discussed in these seven scientific papers, such as disclosure of CSA, poly-victimization, media awareness, self-compassion, or treatments for the persons who committed CSA.

Racine et al. reviewed the differences—Demographic characteristics, abuse characteristics, trauma symptoms, and protective factors—between 64 children who were sexually abused only and 53 poly-victimized children. Their results show a pronounced risk for children who experienced poly-victimization to face challenging familial situations with less protective factors, highlighting the importance of factors impeding the child's developmental ecology. The importance of protective factors, such as self-compassion, is also highlighted in the article of Wekerle et al..

An important issue in terms of the prevention of child sexual abuse is the treatment of (potential) offenders. von Franquè and Briken presented data from a study comparing pedophilic interests, risk factors, responsivity features, and treatment progress in two groups of men: convicted offenders who underwent mandatory treatment and men who were not convicted but participated in voluntary treatment. On the same subject, Fromberger et al. published the study protocol of their clinical trial about a web-based intervention for convicted individuals who sexually abused children, this study will be the first to compare the effectiveness of a guided web-based intervention vs. a placebo condition.

On a societal level, the Frentzen et al. study aimed to address whether the awareness of CSA in the media affects or not the actions of the individuals. Their results show that 62.6% of the participants of their survey were aware of CSA through the media. The authors called for more media bystander interventions.

Finally, Carretier et al. focused on the disclosure of sibling sexual abuse. In their case report, they present vignettes of three girls between 13 and 15 years who disclosed having been abused by their siblings during psychiatric inpatient treatment. The case report illustrates the complexity of sexual abuse and its disclosure, with a specific focus on sibling abuse. Similarly, in the meta-synthesis of Manolios et al., they included 20 articles about the disclosure of CSA to health professionals. Their findings also show how complex disclosure can be, with survivors having a diachronic approach to the experience of disclosure with a feeling of relief and release seen only among the adult participants, long after the disclosure.

These two articles of the collection focus on disclosure (Carretier et al.; Manolios et al.). They highlight how important a systematic search for sexual abuse is in clinical routine. In fact, the search for sexual abuse should be handled in the same way as the search for suicidal ideation by the child & adolescent psychiatrists (CAPs) and other mental health professionals, that is, asking systematic and explicit questions without any ambiguity, but also knowing what to do if a disclosure happens.

Indeed, CAPs must actively and systematically conduct investigations for sexual abuse in their young patients. But, in reality, we believe that this systematic search is a necessary but not sufficient recommendation to give. It is of course necessary that professionals are trained on how to investigate CSA and how to integrate it in their routine clinical practice. Yet, this work also requires a responsibility, both in moral and protective terms, of mental health professionals. Beyond soft skills and empathy, disclosure of CSA requires knowing how to act and how to react. Further research is needed to better understand what allows a child or an adolescent to unburden themselves and reveal the CSA.

Our professional and moral position is that a translation into reality is always necessary after the disclosure of sexual violence. Health professionals to whom a child has disclosed CSA must be able to act and react accordingly to ensure the immediate protection of the child, but also to allow the reparation process and stop or reduce victimization and revictimization risks. We think that relativism and “taking time to elaborate” have no place in front of these disclosures of CSA. Mental health professionals investigating sexual violence should assure beforehand their young patients that if they reveal a CSA, then they would be present to accompany them and that they definitely would know how to act and react concretely to such disclosure.

Keeping the analogy with the search for suicidal ideation, we can conclude that health professionals should consider the disclosure of sexual abuse, even if it occurred a long time ago, as an emergency for the patient, since the consequences of the “failure” of this disclosure could really be harmful and deleterious without an appropriate and immediate reaction from professionals.

## Author contributions

JS, MR, and AR-L wrote the draft of the editorial together. All authors contributed to the article and approved the submitted version.

